# Delivery of Intravenously Administered Antibodies Targeting Alzheimer’s Disease-Relevant Tau Species into the Brain Based on Receptor-Mediated Transcytosis

**DOI:** 10.3390/pharmaceutics14020411

**Published:** 2022-02-14

**Authors:** Toshihiko Tashima

**Affiliations:** Tashima Laboratories of Arts and Sciences, 1239-5 Toriyama-cho, Kohoku-ku, Yokohama 222-0035, Japan; tashima_lab@yahoo.co.jp

**Keywords:** drug delivery into the brain, transendothelium based on receptor-mediated transcytosis, immunotherapy, Alzheimer’s disease, anti-tau and anti-receptor bispecific monoclonal antibodies, Alzheimer’s disease-relevant tau species, temporal-spatial pathological Aβ and tau distribution, interactions between Aβ and tau, tau clearance in microglia, tau clearance in neurons

## Abstract

Alzheimer’s disease (AD) is a neurodegenerative disease that causes memory loss, cognitive decline, and eventually dementia. The etiology of AD and its pathological mechanisms remain unclear due to its complex pathobiology. At the same time, the number of patients with AD is increasing worldwide. However, no therapeutic agents for AD are currently available for definitive care. Several phase 3 clinical trials using agents targeting amyloid β (Aβ) and its related molecules have failed, with the exception of aducanumab, an anti-Aβ monoclonal antibody (mAb), clinically approved by the US Food and Drug Administration in 2021, which could be modified for AD drug development due to controversial approval. Neurofibrillary tangles (NFTs) composed of tau rather than senile plaques composed of Aβ are correlated with AD pathogenesis. Moreover, Aβ and tau pathologies initially proceed independently. At a certain point in the progression of AD symptoms, the Aβ pathology is involved in the alteration and spreading of the tau pathology. Therefore, tau-targeting therapies have attracted the attention of pharmaceutical scientists, as well as Aβ-targeting therapies. In this review, I introduce the implementations and potential of AD immunotherapy using intravenously administered anti-tau and anti-receptor bispecific mAbs. These cross the blood-brain barrier (BBB) based on receptor-mediated transcytosis and are subsequently cleared by microglia based on Fc-mediated endocytosis after binding to tau and lysosomal degradation.

## 1. Introduction

**Figure 1:**
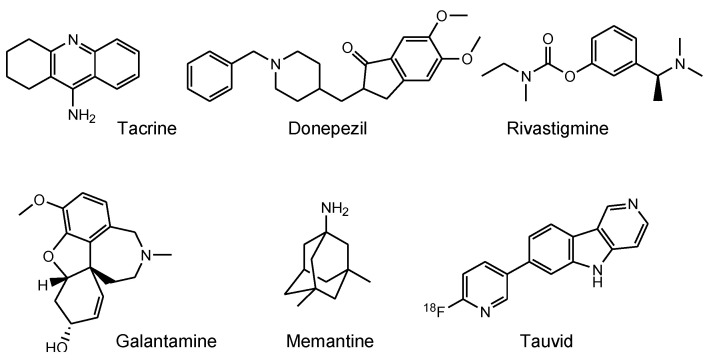
Structures of approved drugs and a tau imaging agent for Alzheimer’s disease.

Medicinal remedies provide long-term health benefits to humans. However, treatments for several important diseases have yet to be developed, including neurodegenerative diseases, such as Alzheimer’s disease (AD) and Parkinson’s disease (PD). Almost all clinical trials for AD, including phase 3 studies using pharmaceutical agents targeting amyloid β (Aβ) and its related molecules, such as β-secretase 1 and γ-secretase, have failed. Despite this, several approved AD drugs do not target Aβ. As a result, many pharmaceutical companies have abandoned efforts to develop AD drugs [1]. Under these circumstances, aducanumab [2], anti-insoluble Aβ fibrils, and anti-soluble Aβ oligomer monoclonal antibody (mAb), was approved by the US Food and Drug Administration (FDA) in 2021. Other existing AD drugs, such as four cholinesterase inhibitors (tacrine, donepezil, rivastigmine, and galantamine) and an *N*-methyl-D-aspartate (NMDA) receptor antagonist (memantine) (Figure 1) [3], have been found to transiently slow down the progression of symptoms; however, they do not lead to definitive therapy. Accordingly, innovative therapeutic agents for AD are expected to be produced as early as possible. An analysis of several clinical trial failures and AD research has suggested alternative strategies for the targeting of tau, in addition to Aβ. The so-called amyloid hypothesis, which states that the accumulation and deposition of oligomeric or fibrillar Aβ peptide are the primary cause of AD, may need to be modified in line with these novel findings. Although Aβ and tau pathologies initially proceed independently, Aβ pathology is involved in the alteration and spreading of tau pathology at a certain point of AD symptom progression [4]. Moreover, the presence of neurofibrillary tangles (NFTs) composed of tau rather than senile plaques composed of Aβ is correlated with AD pathogenesis [5]. Thus, tau clearance in the brain is a promising methodology for treating AD.

**Figure 2:**
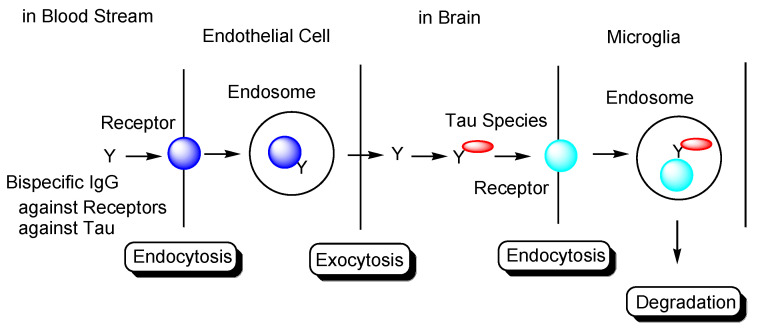
Strategy used to eliminate tau species using anti-receptor and anti-tau bispecific monoclonal antibodies based on receptor-mediated endocytosis and lysosomal degradation in microglia to cure Alzheimer’s disease.

Drug membrane permeability is a serious problem that needs to be addressed in the field of drug discovery and development. The blood-brain barrier (BBB) prevents drugs that target the central nervous system (CNS) from entering the brain. The BBB consists of: (i) a biological barrier based on excretion transporters, such as multiple drug resistance 1 (MDR1), which excludes hydrophobic low-molecular compounds just passing inside the membrane; (ii) a physical barrier based on the tight junctions between the capillary endothelial cells backed by pericytes. Nonetheless, strategies that enable the delivery of substances into the brain across the BBB, based on solute carrier (SLC) transporter-mediated transport or receptor-mediated transcytosis, depending on molecular size and hydrophilicity, have been developed. Several methods for membrane substance permeation exist, including substances that (i) are subject to SLC transporter-mediated transport across the membrane [6], (ii) are transported into cells using cell penetrating peptides (CPPs) [7], (iii) specifically enter cancer cells through receptor-mediated endocytosis based on the enhanced permeability and retention (EPR) effect using nanoparticles [8], (iv) are delivered into the brain based on receptor-mediated transcytosis across the BBB after intravenous administration [9] or (v) across the olfactory epithelium after intranasal administration using insulin as a carrier [10], and (vi) deliver orally administered mAbs as cargo through neonatal Fc receptor (FcRn)-mediated transcytosis across the intestinal epithelium into systemic circulation using enteric nanoparticles [11]. These pharmacokinetic findings are also useful for the design and development of AD drugs. In response to the molecular size and hydrophobicity of drugs used in pharmacological treatment, the methodology of drug design and pharmacokinetic trajectory vary. CNS drugs, such as AD therapeutic agents, must cross the BBB to elicit their activity. It is difficult for anti-tau mAbs to cross the cell membrane, because they are large and hydrophilic molecules. In this perspective review, I introduce immunotherapy using intravenously administered anti-tau mAbs to be delivered into the brain based on receptor-mediated transcytosis, particularly using a bispecific strategy (Figure 2). Furthermore, to accomplish well-defined mAb drug design, I refer AD pathological mechanisms caused by factors such as Aβ and tau.

## 2. Discussion

### 2.1. Alzheimer’s Disease

AD is a progressive neurodegenerative disorder that leads to synapse loss, neuronal cell death, and eventually dementia, first reported by Alois Alzheimer in 1906. The number of American AD patients aged 65 and older in 2021 is 6.2 million, which is estimated to rise to 13.8 million by 2060 [12]. Worldwide, approximately 50 million people were reported as having dementia in 2020, 60–70% of whom are also associated with AD. This is expected to rise to 82 million in 2030 and 152 million in 2050 [13]. The social and economic impacts of AD, including treatment by care takers and health care workers, patient dignity, and medical bills, are important issues. However, many aspects of AD, including its etiology, progression, and treatment, remain unknown due to its multifactorial and complex mechanisms. The structure of AD based on structuralism advocated by Lévi-Strauss, which systematically and comprehensively regulates AD pathogenesis and pathology based on biological and physical components, remains poorly understood.

Senile plaques composed of Aβ and NFTs comprised of hyperphosphorylated tau are widely accepted hallmarks of AD. This implies that Aβ and tau play important roles in the onset and progression of AD. Currently, AD drugs that focus on Aβ modulators or tau modulators have been developed. However, they are considered unlikely to be able to achieve the desired outcomes in many cases. Therefore, a well-defined drug design based on the biological and physical structures that result in AD pathology needs to be developed.

### 2.2. Amyloid Hypothesis

Although the mechanism of AD onset and progression remains unclear, the amyloid hypothesis is widely accepted to explain the primary cause of AD in drug development. According to this theory, Aβ, which is mainly divided into Aβ (1–42) and Aβ (1–40) due to amino acid sequence length, is cleaved by β-secretase (β-site APP cleaving enzyme 1 (BACE1)) and then by γ-secretase from amyloid precursor protein (APP) [14]. The population of Aβ (1–42) is less than that of Aβ (1–40). However, Aβ (1–42) is supposed to aggregates more easily than Aβ (1–40), subsequently eliciting neurotoxicity. Aβ assembly species structurally mutate from unfolded monomers, folded monomers, oligomers, and protofibrils to fibrils. The structures and locations of the deposited Aβ differ.

Senile plaques are believed to be the main factor in the pathology of AD. Although Aβ modulators, such as BACE1 inhibitors, γ-secretase inhibitors, and anti-Aβ Abs, have been developed to date, only aducanumab has been clinically approved for use by the FDA. Despite this, therapeutic approaches to AD treatment using Aβ modulators have been unsuccessful. Nonetheless, the amyloid hypothesis is believed to hold due to APP mutations causing familial AD accounts for 5 to 10% of total AD, although the involvement of tau mutations has not yet been observed in familial AD, in contrast to other tauopathies, such as frontotemporal dementia and parkinsonism linked to chromosome 17 (FTDP-17). Three known genes with mutations associated with familial AD are *presenilin 1* (*PSEN1*), *presenilin 2* (*PSEN2*), and *APP*. Presenilin 1 (PS1) and presenilin 2 (PS2) form the catalytic core component of the γ-secretase complex. Many APP mutations have been reported. Among them, the Osaka mutation, with APP E693del, did not form Aβ protofibrils, senile plaques, and NFTs, but formed Aβ oligomers and phosphorylated tau in transgenic mice with APP E693del (Osaka). In this in vivo assay, most AD pathologies were reproduced, even without NFT formation. Moreover, Aβ oligomer accumulation, NFT formation, and AD pathologies, such as synapse loss, neuronal loss, and memory impairment, were observed in double transgenic mice with APP E693del (Osaka) and human tau [15]. These findings suggest that Aβ monomers and/or oligomers induce AD pathologies by interacting with AD-relevant tau species through direct and/or indirect interactions between Aβ and tau. Nevertheless, Aβ protofibrils and other non-oligomeric species may elicit neurotoxicity through ways other than Aβ monomers and oligomers, as the pathology of AD is known to be established via complex and intricate processes.

AN-1792 is a synthetic full-length Aβ peptide with QS-21 adjuvant as a vaccine to produce anti-Aβ Abs, which was evaluated for its efficacy in clinical trials against mild to moderate AD patients. Surprisingly, as a result, AD dementia in some patients was found to progress, although their senile plaques in the brain disappeared [16]. However, Aβ oligomers were not completely eliminated [17]. Based on these results, several conclusions were suggested: (i) factors different from Aβ, such as tau, may act as causative substances; (ii) Aβ oligomers rather than senile plaques may evoke AD pathology; (iii) treatment was delayed after the onset of AD symptoms started to appear. This suggests that AD could not be cured, even though senile plaques were eliminated. Therefore, alternative therapeutic approaches should be developed. In fact, the amyloid hypothesis was modified by considering the interaction between Aβ oligomers and tau oligomers after a certain stage of AD.

### 2.3. Tau Proteins

**Figure 3:**
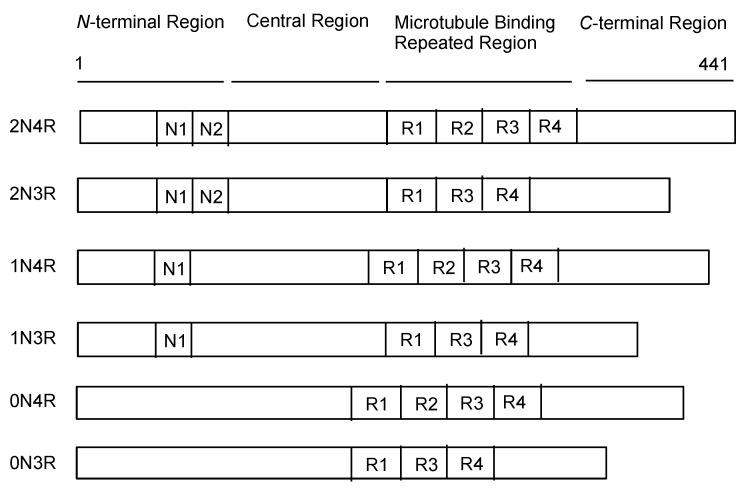
Schematic representation of the protein structures of tau.

Tau protein [14,18] is encoded by the microtubule-associated protein tau (MAPT) gene on chromosome 17. According to the splicing pattern, there are six tau protein isoforms (0N3R, 0N4R, 1N3R, 1N4R, 2N3R, and 2N4R) composed of four regions: the *N*-terminal domain, proline-rich domain (PRD), microtubule-binding domain (MBD), and *C*-terminal domain. While the 3-repeat (3R tau) isoform is exclusively expressed in the fetal brain, the 4-repeat (4R tau) and 3R tau isoforms are evenly expressed in the adult brain (Figure 3).

Physiological tau proteins play a vital role not only in the stabilization of microtubules in axions via their MBDs, but also in other biological functions [14]. When tau is phosphorylated, tau becomes detached from the microtubules, resulting in microtubule instability. Currently, there are at least 30 phosphorylation sites among 85 residues with hydroxyl groups in a tau protein (2N4R). Mathematically, the number of multiphosphorylated tau proteins is a myriad. At least eight sites are phosphorylated in AD pathological tau, whereas 2 to 3 sites are phosphorylated in normal physiological tau [19]. Moreover, tau is involved in other post-transcriptional modifications, such as acetylation, glycosylation, ubiquitination, and truncation. Thus, a variety of tau species exist with respect to isoforms and post-transcriptional modification, which renders the pathological mechanism unclear.

Phosphorylated tau (p-tau) monomers and p-tau oligomers exhibit neurotoxicity, intercellular distribution, seeding, and aggregation, which are different from physiologically intact tau proteins. Tau assembly species structurally mutate from phosphorylated monomers, phosphorylated oligomers, and filaments, such as paired helical filaments (PHF), to tangles. It has been pointed out that the population of NFTs is correlated with the pathogenesis of AD dementia, while that of Aβ plaques is not [5]. Therefore, it is suggested that tau plays a vital role in AD pathology, although Aβ is also involved in it due to the existence of familial AD accompanied by mutations in APP, presenilin 1, or presenilin 2.

NFT formation in the neuronal cell body is thought to be associated with the progressive AD process due to detoxification [20,21]. NFTs were thoroughly inactive, such that they remained as extracellular ghost tangles, even though the host neurons died and disappeared. p-Tau oligomers were found to be the most toxic species. Thus, PHFs may also form as a result of the progressive AD process, although they elicit toxicity as physical substances. Inactive p-tau aggregates were not soluble, even with detergents. Accordingly, approaches using Abs neutralizing soluble p-tau oligomers and p-tau monomers as their composition are promising methods for AD therapy [22].

### 2.4. Aβ and Tau Pathologies

#### 2.4.1. Temporal-Spatial Pathological Aβ and Tau Distribution in the Brain

In 1989, Ihara pointed out that AD progression irreversibly began when tau was distributed until the temporal lobe across the collateral sulcus via the hippocampus and the parahippocampal gyrus [23]. More recently, it was revealed that the temporal-spatial behavior of Aβ and tau proteins changes with respect to formation and distribution. Aβ pathology and tau pathology were performed independently. However, at a certain point, Aβ pathology began to assist tau pathology to enhance the pathological process of AD [4,24]. This pathophysiological hypothesis is consistent with familial AD due to APP mutations. At the onset of dementia, Aβ is already saturated in the brain. Even though Aβ was removed, dementia proceeded without Aβ, most likely due to the formation of the pathological tau seeds, which duplicated and transmitted cell-to-cell, via interactions with Aβ at that stage, propagating pathological tau as a result. As widely accepted among medicinal chemists, seed crystals initiate crystallization during organic synthesis. When washing using glass vessels is insufficient, crystallization of the same compounds proceeds by the remaining crystals without the addition of seed crystals. Therefore, the clearance of tau to avoid its propagation may be useful for the treatment of AD.

Tauvid, approved by the FDA in 2020, was the first and only agent to image tau pathology, particularly NFTs, using positron emission tomography (PET) (Figure 1). Tau imaging enables the observation of the distribution of tau in the brain of AD patients, allowing to predict AD symptom progression by comparing data on the correlation between the temporal-spatial distribution of pathological tau and the pathology of AD.

#### 2.4.2. Cell-to-Cell Pathology Transmission

Knowledge of tau trajectory is useful for anti-tau mAb drug design. Some inspired readers should suggest better ideas. Tau pathology is thought to be template-dependent progressive cell-to-cell and occur via regional transmission in a prion-like manner. The physical and pathological properties of tau are inherited from the original tau seeds, which form disease-specific tau pathology based on tau strains in AD, Pick’s disease (PiD), progressive supranuclear palsy (PSP), and corticobasal degeneration (CBD) [25], appearing to belong to unelucidated respective certain strains. It is likely that extracellular tau is released from a donor neuron into the brain interstitial fluid (ISF) and transferred into the synaptically connected recipient neurons across the plasma membrane through endocytosis rather than into neurons of spatial proximity. The precise mechanisms of such transportation processes during the development of tauopathy remain a topic of controversy [18,26,27,28].

**Figure 4:**
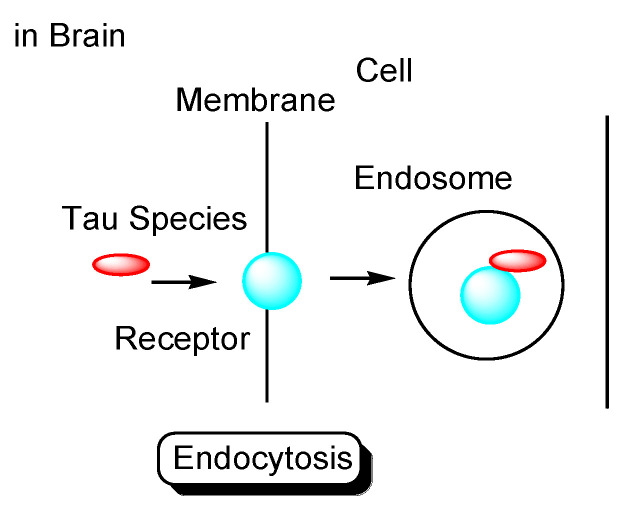
Tau species enter the cells based on endocytosis such as receptor-mediated endocytosis.

**Figure 5:**
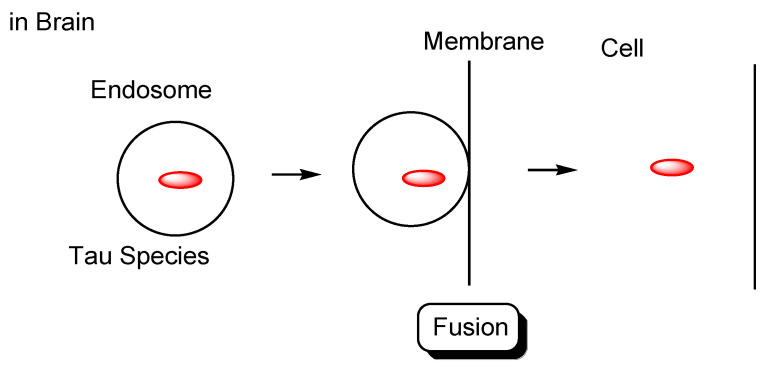
Tau species in endosomes enter the recipient cells based on membrane fusion.

Tau species enter neuronal cells either: (i) through several internalization mechanisms based on endocytosis, such as receptor-mediated endocytosis (Figure 4), or (ii) through exosome fusion to the plasma membrane of recipient cells (Figure 5). Monomeric tau, such as monomeric tau p301S covalently labeled with Dylight, a fluorescent dye, or with pHrodo, a pH-sensitive dye, was internalized into neurons through dynamin-dependent endocytosis and micropinocytosis, using human stem cell-derived neurons. On the other hand, aggregated tau, such as aggregated tau p301S covalently labeled with Dylight or pHrodo, was internalized through dynamin-dependent endocytosis. Monomeric and aggregated tau in early endosomes (pH 6.5) was degraded in lysosomes (pH 4.5) as endosome maturation via late endosomes (pH 5.5). These processes of tau entry occur not only in AD pathological neurons but also in physiologically normal neurons [29].

**Figure 6:**
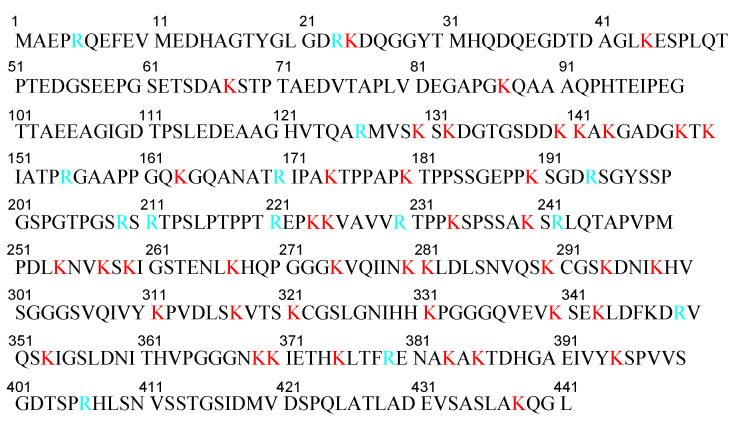
Amino acid sequence of human tau441 (2N4R). Lysines are shown in red and arginines are shown in light blue.

Second, Alexa Fluor^TM^-labeled tau oligomers derived from AD and DLB brains were transported into cortical neurons isolated from C57BL/6 mice through heparan sulfate proteoglycan (HSPG)-induced, clathrin- and caveolae-independent endocytosis, and remained in endosomes from early endosomes to lysosomes. In contrast, labeled tau oligomers derived from PSP brains were transported through other pathways [30]. Positively charged CPPs, short amino acid oligomers (5–30 residues), such as TAT and R8, electrostatically interact with negatively charged HSPGs to induce endocytosis [31]. Accordingly, this suggests that p-tau (352 residues (0N3R)–441 residues (2N4R)) is able to enter neurons through HSPG-induced endocytosis based on electrostatic interactions, since it has as many as 44 lysine residues and 14 arginine residues in the 441 amino acid sequence (Figure 6). Tau and HSPG are able to interact due to the fact that heparin acts as a core for tau seeding and aggregation [32,33].

Third, low-density lipoprotein receptor-related protein 1 (LRP1) is involved not only in tau endocytosis, but also in the distribution of tau in the brain. It is worth noting that this distribution was diminished in LRP1 knockout mice [34].

Fourth, M1 and M3 muscarinic receptors were correlated with tau entry into neurons. Physiologically normal tau stabilizes neurites, while pathological tau induces neurodegeneration, leading to cell death in cerebellar neuronal cultures. The uptake of tau in cerebellar neurons was inhibited by an M1 antagonist, such as pirenzepine, but not by M2 antagonists, such as AF-DX116, or an M2/M4 antagonist, such as pertussis toxin. Tau uptake was increased approximately 19-fold in CHO cells transfected with M1 or M3, and 31.5-fold in CHO cells transfected simultaneously with both M1 and M3 by immunoblot analysis using anti-human tau, compared to non-transfected CHO cells [35].

The internalization cases presented here act through the endocytosis pathway, leading to degradation in lysosomes, although some of the endocytosed tau seeds may be transported and localized in the cytoplasm by endosomal escape, or may alternatively be recycled back to the outside based on the fusion to the plasma membrane regulated by Rab7A. Accordingly, the cell-to-cell transmission of tau seeds would not be accomplished as long as tau seeds are subject to such clearance in lysosomes. Tau, which contains lysine residues that accept proton influents through vacuolar adenosine triphosphatases (V-ATPases), may be released into the cytosol from endosomes due to membrane bursting, based on the osmotic gap due to the proton sponge effect [8]. On the other hand, its direct translocation into cells across the membrane has also been suggested. In contrast to CPPs, it is difficult for tau to be internalized through direct translocation due to its size. Subsequently, tau endosomal escape into the cytosol has been found to play an important role not only in tauopathy progression, but also in physiological tau activity. Even though duplicated tau species are formed in endosomes in a prion-like manner, they are enzymatically degraded into pieces in lysosomes. In fact, tau is released from endosomes, although the mechanism by which this occurs remains unclear [36].

**Figure 7:**
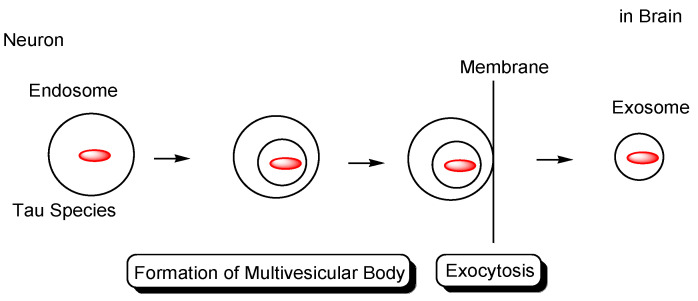
Tau in exosomes (40–100 nm in diameter) secreted from neurons.

**Figure 8:**
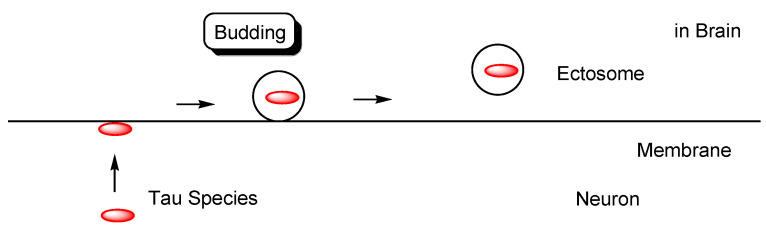
Tau in ectosomes (50–1000 nm in diameter) secreted from the membrane of neurons, independently on the endolysosomal machinery.

**Figure 9:**
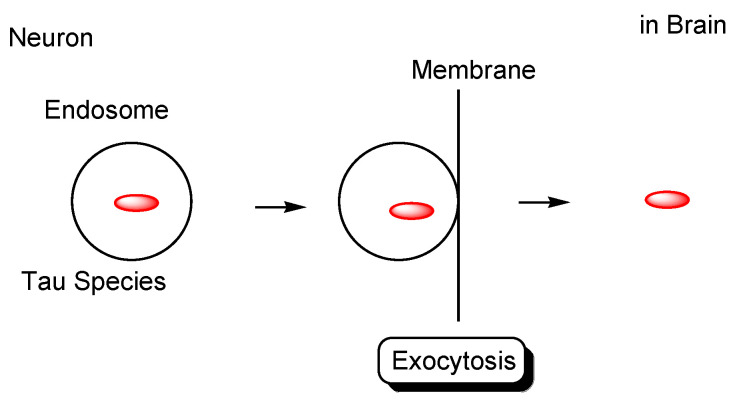
Tau secreted from neurons based on the endolysosomal machinery.

**Figure 10:**
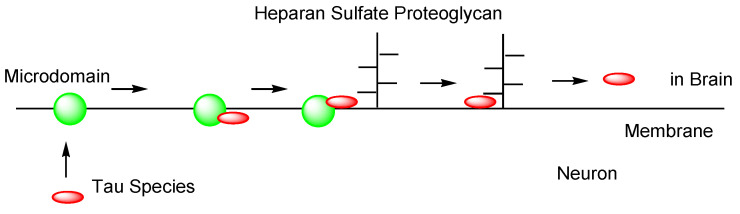
Tau secreted from neurons through an unconventional non-vesicular mechanism.

However, as another internalization mechanism different from endocytosis, tau in extracellular exosomes can be released into the neuronal cytoplasm after membrane fusion in recipient cells. In this case, there is no problem involved in endosomal escape because tau is not present in endosomes. In fact: (i) some tau proteins are present in exosomes secreted from cells (Figure 7) [18]. Accordingly, tau secretion mechanisms from donor cells are also important for investigating substantive tau transmission, in addition to the internalization mechanism in recipient cells. Interestingly, ectosomes (50–1000 nm in diameter) are extracellular vesicles that do not depend on the endolysosomal machinery to produce exosomes (40–100 nm in diameter) [37]. They budded out and were cut off from the plasma membrane; (ii) some tau proteins are present in these ectosomes (Figure 8). Nevertheless, it was revealed that free tau that was not in exosomes was secreted from donor neurons through (iii) endosomal fusion to the plasma membrane regulated by Rab7A (Figure 9) or (iv) direct translocation, despite its large size [38]. Surprisingly, it was reported that over 99% of tau oligomers are secreted in a membrane microdomain- and HSPG-dependent unconventional vesicular-free mechanism in mouse N2A neuroblastoma cell line, approximately 80% of which are characterized as dimers, trimers, or tetramers (Figure 10) [38]. Thus, anti-tau Abs in ISF can bind to tau to block cell-to-cell transmission. As reference examples, at the molecular level, cytoplasmic full-length TAT (101 amino acids) was attached to anionic phosphatidylinositol 4,5-bisphosphate (PI(4,5)P_2_) in the inner leaflet of the lipid bilayer through the cationic region (residues 49–57), inserting its hydrophobic Trp11 residue for itself to be buried in the membrane, and thereby permeated to the extracellular space [39]. Moreover, cytoplasmic fibroblast growth factor 2 (FGF2) was attached to anionic PI(4,5)P_2_ in the inner leaflet of the lipid bilayer, permeated through the membrane based on the microdomain after oligomerization, and interacted with HSPGs in the outer leaflet of the lipid bilayer to be released [40]. Similarly, cytoplasmic tau was attached to anionic PI(4,5)P_2_ and phosphatidylserine in the inner leaflet of the lipid bilayer, went through microdomain-mediated membrane permeation after oligomerization, bound to HSPGs in the outer leaflet of the lipid bilayer, and was thereby liberated to the extracellular space in its free form [18,39]. Microdomains are composed of lipid rafts, cholesterol, and sphingomyelin.

**Figure 11:**
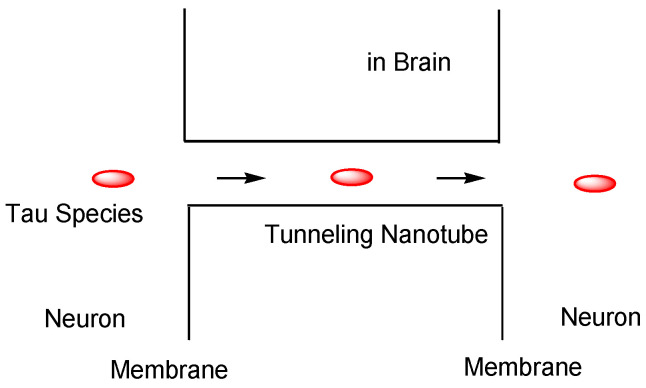
Tau cell-to-cell transportation through tunneling nanotubes.

Furthermore, (v) tau may be transmitted cell-to-cell through tunneling nanotubes (50–700 nm in diameter, 20–100 μm length) (Figure 11) [41]. Although tau cannot diffuse in the extracellular space, tunneling nanotubes remain controversial in vivo, despite being recognized in cell lines [36].

mAb drugs exhibit high selectivity against antigens. Thus, conformation-selective mAbs targeting pathological tau may suppress tau toxicity and cell-to-cell pathology transmission without disturbing constitutive tau homeostasis and function due to unbinding to physiological tau.

### 2.5. Tauopathies

Tauopathy is a general term for neurodegenerative diseases with NFTs composed of tau proteins that originally spread via cell-to-cell transmission process and duplicated to tau seeds in recipient cells [25]. It is well known that disease-specific tau pathology is based on tau strains in AD, PiD, PSP, and CBD. Brain-derived tau oligomers from AD and dementia with Lewy bodies (DLB) were internalized through HSPG-mediated endocytosis using cortical neurons isolated from C57BL/6 mice, while those from PSP were internalized through not only HSPG-mediated endocytosis but also through alternative mechanisms [30]. Some tauopathies are associated with tau gene mutations. Thus, it is thought that arbitrary tau strains are doomed to induce the corresponding tauopathy-based structuralism by Lévi-Strauss. Tau oligomers composed of 3R and 4R isoforms were aggregated through the seeding of PSP brain-derived tau oligomers with 3R and 4R monomers after the addition of monomeric 3R and 4R tau in phosphate-buffered saline. However, pathologically, only NFT composed of 4R tau was formed in the PSP. It has been suggested that 4R tau is more toxic than 3R tau in PSP. Toxic 4R tau isoforms may be selectively packed into NFTs as inclusion bodies in the detoxifying process [42]. NFTs in AD are composed of 3R and 4R tau. 4R tau may be as toxic as 3R tau, particularly in oligomeric forms, in AD. Moreover, 2N4R tau is more toxic than 1N4R tau in neurons [43].

### 2.6. Interactions between Aβ and Tau That Induce Neurotoxicity

Aβ and tau are recognized as the major factors driving the pathology of AD. They are thought to interact with each other during pathological processes. After incubating Aβ monomers for 5 days in 10-day-old organotypic hippocampal slices prepared from rat pups, Aβ oligomers were formed. Interestingly, the phosphorylation of tau at Ser396/Ser404 binding to PHF-1 Ab and at Thr231/Ser235 binding to AT180 Ab were augmented by more than two-fold after Aβ incubation, compared to no Aβ incubation as the control, while those at Ser199/Ser202 binding to AT-8 Ab and at Thr212/Thr217/Ser214 binding to AT100 Ab did not change [44]. This result indicates that Aβ affects the profiles and levels of tau phosphorylation.

#### 2.6.1. Cyclin-Dependent Kinase 5 (CDK5) as a Matchmaker between Aβ and Tau

Glutamatergic tripartite synapses, composed of presynaptic neurons, postsynaptic neurons, and astrocytes, play an integrative role not only in neural networks for learning and memory, but also in AD pathogenesis and pathology [45]. Synaptic *N*-methyl-D-aspartate receptors (sNMDARs), constituted of GluN1/GluN1/GluN2A/GluN2A subunits as major [46,47] or GluN1/GluN1/GluN2A/GluN2B subunits as minor [48], are located inside the synapse of the postsynaptic neuron and exhibit neuroprotective activity, whereas extrasynaptic *N*-methyl-D-aspartate receptors (eNMDARs) consisting of GluN1/GluN1/GluN2B/GluN2B subunits [46,47] are located outside the synapses of postsynaptic neurons and exhibit neurotoxic activity.

Excess Glu molecules in the synaptic cleft were sufficient to activate the eNMDARs. Aβ induces eNMDARs to influx calcium ions into neurons. Calcium ions that flow through activated eNMDARs cleave p35 into p25 and p10 by calpain. The resultant p25/CDK5 complex mediates tau phosphorylation [49].

#### 2.6.2. Fyn as a Matchmaker

At the postsynaptic site of the glutamatergic tripartite synapse, NMDAR with GluN2B is involved in tau phosphorylation via Fyn [50], which is a cytoplasmic tyrosine kinase. Aβ oligomers bind to cellular prion protein (PrPc) and subsequently activate Fyn in the NMDAR-postsynaptic density protein-95 (PSD95)-Fyn complex at the postsynaptic site [51]. The resulting activated Fyn phosphorylates the GluN2B of sNMDAR at Tyr1472 and GluN2B of eNMDAR at Tyr1336 [52] to stabilize the NMDAR-PSD95-Fyn complex location at the membrane, which enhances glutamatergic excitotoxicity by Aβ oligomers. Moreover, p-tau bound to Fyn and/or physiological tau bound to it carried it to dendritic spines [53], and formed the NMDAR-PSD95-Fyn-p-tau complex to enhance glutamatergic excitotoxicity due to alteration of complex conformation by tau. Intriguingly, Fyn did not form a complex with NMDAR in transgenic mice expressing truncated tau and tau-/- mice [53,54]. Tau can be phosphorylated in such complexes. In particular, the Fyn-tau interaction was increased by phosphorylation at serines where AT8 Abs bind. AT8 Ab-binding phosphorylated tau was localized postsynaptically 7-fold greater than before NMDAR activation via Aβ oligomer formation in an in vitro assay detected by electron microscopy using embryonic day 18 rat hippocampal slices. By contrast, tau was phosphorylated at the sites where PHF-1 Ab (against p-Ser396/p-Ser404) or AT180 Ab (against p-Thr231/p-Ser235) bind after Aβ oligomer formation, and tau was phosphorylated at the sites where AT8 Ab (against p-Ser199/p-Ser202) or AT100 Ab (against p-Thr217/p-Ser214) bind after NMDAR activation [44]. As a result, p-tau and Aβ oligomers synergistically exacerbate AD pathology. On the other hand, Fyn activated via phosphorylation at Tyr416 by Pyk2 phosphorylated tau at Tyr18 [51]. It has been suggested that the phosphorylation of tau at Tyr18 is necessary for NMDAR-mediated excitotoxicity [55]. However, the mechanisms underlying this action remain elusive, and must be clarified in future studies. Although p-tau binding to AT8 and AT100 was not increased by the addition of Aβ, it is known that such sites are phosphorylated in the late stage of AD [44]. Therefore, the pathological mechanisms in the late stage were assumed to be different from those in the early stage.

Physiological tau monomers exist in the dendritic cytoplasm, as well as in the microtubules. Dendritic tau recruits Fyn to the NMDAR-PSD95 complex. Thus, the source of such dendritic tau is not found in the extracellular region but in the intracellular region due to local tau mRNA translation or dissociation from microtubules. Moreover, it was revealed that p-tau constitutively present in normal neurons was located in dendrites and near synapses rather than in axons. While p-tau binding to PHF-1 posed somatodendric localization in primary neurons isolated from embryonic day 18 rats, p-tau binding to AT180 and AT8 posed dendric localization [44].

#### 2.6.3. Ca^2+^-Dependent Calmodulin Kinase IIα (CaMKIIα) as a Matchmaker

The Aβ*56 oligomer (4.8–5.7 nm; diameter, 56 kDa) enhanced Ca^2+^ inflow by binding to sNMDAR. An increase in the intracellular Ca^2+^ concentration activated CaMKIIα and subsequently induced approximately 2.7-fold higher tau phosphorylation at Ser202 and Ser416 in 7-month-old Tg2576 mouse forebrains compared to non-transgenic littermates. Aβ dimers and trimers do not exhibit this tau phosphorylation pathway. In addition, Aβ*56 does not activate Fyn [56,57].

#### 2.6.4. GSK3β as a Matchmaker

In terms of post-synaptic density, Aβ induces sNMDAR-mediated tau phosphorylation at Ser396 by GSK3β [58]. In contrast, the eNMDAR-PSD95-Fyn-p-tau complex involved in Aβ oligomers activates GSK3β and CDK5 [44]. Aβ oligomers bind to α2A adrenergic receptors and enhance GSK3β-mediated tau phosphorylation in mice [59].

#### 2.6.5. c-Jun N-Terminal Kinase (JNK) as a Matchmaker

Soluble Aβ (1–42) monomers bind to β2 adrenergic receptors that belong to G protein-coupled receptors and induce JNK tau phosphorylation at Ser214 [60].

### 2.7. Implementation of mAbs Targeting AD-Relevant Tau Species

#### 2.7.1. Conformation-Selective Anti-Tau mAbs Block Cell-to-Cell Transmission of Tau Pathological Seeds

It has been suggested that tau seeds drive the interneuronal propagation of tau from one area of the brain to another in AD patients over time via prion-like mechanisms. Eliminating arbitrary tau species that become seeds is a strategy to inhibit AD progression using mAbs. Several mAbs against p-tau species, particularly monomers and oligomers, have been developed [22]. Furthermore, a number of mAbs selective to tau forms and/or conformations have been developed. DMR7 and SKT82 are mAbs that selectively bind to the misfolded pathological conformation of tau. DMR7 showed EC_50_ values of 0.10 ± 0.01 nM for AD-tau extracted from postmortem human brain tissue, 0.46 ± 0.32 nM for AD-tau seeded recombinant tau preformed fibrils (AD-P1 PFFs) expressed in BL21(DE3)RIL Escherichia coli, and 12.0 ± 7.9 nM for tau monomer through sandwich ELISA measures. On the other hand, SKT82 showed those of 0.17 ± 0.03 nM for AD-tau, 2.38 ± 1.12 nM for AD-P1 PFFs, and 4.13 ± 3.74 nM for tau monomer. These two mAbs bound extracellularly to tau seeds, such as AD-P1 PFFs, and inhibited their uptake into cells and subsequent seeded fibrillization or aggregation based on internalized seeds in in vitro tests using primary neurons. This may be due to blocking of tau binding to receptors, such as LRP1, M1 and M3 muscarinic receptors, or heparan sulfate proteoglycans. Furthermore, SKT82 exhibited more effective tau pathology inhibition than DMR7 in slice cultures and the ipsilateral hippocampus in vivo. SKT82 (mouse IgG2b isotype) binding to AD-tau interacted with FcR on the membrane of microglia and was cleared greater than DMR7 (mouse IgG1 isotype) binding to AD-tau in a murine model. DMR7 does not interact with microglia [61].

Tau clearance in lysosomes was conducted through FcγR-mediated endocytosis after binding to the corresponding Abs in primary mouse microglial cultures [62]. FcγRI, FcγRIIa, FcγRIIb, and FcγRIIIa are upregulated in AD microglia [63]. It has been reported that human microglia do not induce an increase in inflammation by the tau-Ab complex [64], although reactive microgliosis (a particular state of inflammatory microglia) is suggested to be one of the primary causes of neurodegenerative diseases. Thus, FcR-mediated endocytosis, particularly in microglia, is a promising strategy for tau clearance using anti-tau antibodies.

#### 2.7.2. Clinical Trial for Anti-Tau mAbs

Many clinical trials for Abs targeting Aβ and pharmaceutical agents modulating molecules related to Aβ have been unsuccessful, except for aducanumab as an anti-Aβ Ab. Accordingly, tau, instead of Aβ, has been investigated clinically (Table 1).

Active immunotherapy will result in the development of Abs based on the immune system. AADvac1 (tau 294-305; KDNIKHVPGGGS) or ACI-35 (pSer396 and 404) as active tau vaccines could neutralize tau species. Phase 1 (NCT02031198) [65] and phase 2 (NCT02579252) [66] clinical trials using AADvac1 have been completed, wherein AADvac1 exhibited a safety profile in patients with mild-to-moderate AD and induced Ab titers. Phase 1 and 2 clinical trials for AD (NCT04445831) using ACI-35 are currently ongoing.

For passive immunotherapy, LY3303560 (zagotenemab, a humanized anti-tau Ab) and BIIB076 (a human IgG1 Ab against tau), were tested in phase 1 clinical trials for AD (NCT03019536 and NCT03056729, respectively). Lu AF87908 is a humanized IgG1 Ab against tau and is currently in a phase 1 clinical trial for AD (NCT04149860).

BIIB092 (gosuranemab, a humanized IgG4 Ab against tau) was evaluated for AD in a phase 2 trial (NCT03352557). Moreover, ABBV-8E12 (tilavonemab), a humanized IgG4 Ab against tau aggregates, has been developed for the treatment of PSP and AD. Intravenously administered ABBV-8E12 has been investigated in a phase 2 clinical trial for AD (NCT03712787).

E2814, a humanized IgG1 Ab against tau, has been conducted in phases 1 and 2 for AD (NCT04971733). Phase 2 clinical trials using JNJ-63733657 (a humanized anti-tau Ab) for cognitive dysfunction (NCT04619420) and UCB0107 (Bepranemab, a humanized IgG4 Ab against tau 235-250) for AD (NCT04867616) are currently being conducted.

RO7105705 (semorinemab) is an IgG4 antibody against tau. Semorinemab was evaluated in a phase 2 clinical trial in prodromal to mild AD and was found to surprisingly not be likely to improve outcomes (NCT03289143) [67]. Nonetheless, phase 2 tests have been performed for moderate AD (NCT03828747).

No clinical trials using anti-tau antibodies in AD have led to phase 3 trials. Thus, alternative approaches using anti-tau Abs should be conducted using a well-designed strategy based on absorption, distribution, metabolism, and excretion (ADME). Intravenously administered Abs must cross the BBB and be metabolized together with the captured tau species. Clinically approved anti-Aβ Ab, aducanumab, has been shown to cross the BBB.

**Table 1:** Summary of clinical trials focusing on anti-tau Abs described in this review.

#	Administrated Drug	Formulation /Co-Administrated Drug	Disease	Sponsor	Phase	Study Start Date	Study Completion Date	ClinicalTrials.gov Identifier (Accessed on 25 December 2021)	Status	References
(i)	AADvac1	An active tau vaccine (tau294–305)	AD	Axon Neuroscience SE	Phase 1	January 2014	December 2016	NCT02031198	Completed	[68]
(ii)	RG7345	A humanized Ab against tau pS422	Healthy Volunteer	Hoffmann-La Roche	Phase 1	January 2015	October 2015	NCT02281786	Completed	–
(iii)	RO7105705 (Semorinemab)	An IgG4 Ab against tau	Mild-to-Moderate AD	Genentech, Inc.	Phase 1	June 2016	June 2017	NCT02820896	Completed	–
(iv)	LY3303560 (Zagotenemab)	A humanized Ab against tau/Florbetapir F18	AD	Eli Lilly and Company	Phase 1	January 2017	June 2019	NCT03019536	Completed	–
(v)	BIIB076	A human IgG1 Ab against tau	AD	Biogen	Phase 1	February 2017	March 2020	NCT03056729	Completed	–
(vi)	Lu AF87908	A humanized IgG1 Ab against tau	AD	H. Lundbeck A/S	Phase 1	September 2019	May 2021	NCT04149860	Recruiting	–
(vii)	AADvac1	An active tau vaccine (tau294–305)	Mild AD	Axon Neuroscience SE	Phase 2	March 2016	June 2019	NCT02579252	Completed	[69]
(viii)	ACI-35	An active tau vaccine (pSer396 and 404)	AD	AC Immune SA	Phase 1/2	July 2019	October 2023	NCT04445831	Recruiting	–
(ix)	E2814	A humanized IgG1 Ab against tau	AD	Eisai Inc.	Phase 1/2	June 2021	April 2024	NCT04971733	Recruiting	–
(x)	RO7105705 (Semorinemab)	An IgG4 Ab against tau	Prodromal to Mild AD	Genentech, Inc.	Phase 2	October 2017	January 2021	NCT03289143	Completed	[70]
(xi)	BIIB092 (Gosuranemab)	A humanized IgG4 Ab against tau	AD	Biogen	Phase 2	May 2018	August 2021	NCT03352557	Active, not recruiting	–
(xii)	RO7105705 (Semorinemab)	An IgG4 Ab against tau	Moderate AD	Genentech, Inc.	Phase 2	January 2019	October 2023	NCT03828747	Active, not recruiting	–
(xiii)	ABBV-8E12	A humanized IgG4 Ab against tau aggregates	Early AD	AbbVie	Phase 2	March 2019	July 2021	NCT03712787	Active, not recruiting	–
(xiv)	JNJ-63733657	A humanized Ab against tau	Cognitive dysfunction	Janssen Research & Development, LLC	Phase 2	January 2021	March 2025	NCT04619420	Recruiting	–
(xv)	UCB0107 (Bepranemab)	A humanized IgG4 Ab against tau235–250	AD	UCB Biopharma SRL	Phase 2	June 2021	November 2025	NCT04867616	Recruiting	–
(xvi)	intravenous mAbs	Bispecific mAbs against tau and TrR	AD						Under analysis in Tashima lab	–

### 2.8. Possibility and Effective Use of mAbs Targeting AD-Relevant Tau Species

#### 2.8.1. Lympatic and Immune System in the Brain

The brain lacks a lymphatic system. Instead, a glymphatic system based on cerebral small blood vessels, perivascular cavity, and glial cells develops and eliminates Aβ from the CNS via the bulk flow of cerebrospinal fluid (CSF) from the periarterial cavity to the perivenous cavity via intercellular space, caused by blood vessel pulsation and aquaporin-4 (AQP4) [68]. Sleep disruption promotes tau pathology [69].

Nevertheless, clearance mechanisms are assumed to be performed by brain cells, similar to phagocytosis by immune cell macrophages. Microglia play an important role in the brain. In fact, tau degradation in microglia was found to be enhanced by MC1 (anti-tau mAb) in an Fc-dependent manner [70]. Therefore, FcR-mediated endocytosis by microglia and successive lysosomal degradation may be a promising strategy for extraneuronal tau clearance using anti-tau Abs.

It has been shown that tau-Ab complexes enter neurons. MAb86 (anti-tau/pS422 Ab) bound to lipid raft-associated tau/pS422 on the surface of neurons was reported to be likely to be endocytosed and degraded in lysosomes in AD model TauPS2APP mice [71]. In addition, Dylight-labeled aggregated tau-Ab complexes were internalized to be detected in iPSC-derived human neurons, although its entry mechanism remains unknown [29]. Furthermore, 4E6G7 (anti-tau/pSer396/pSer404 Ab) labeled with ^125^I or Alexa Fluor 568 has been reported to enter neurons in brain slice cultures from hTau/PS1 transgenic mice via clathrin-dependent FcγII/IIIR-mediated endocytosis or fluid phase endocytosis in the presence of both intracellular and extracellular tau aggregates. Ab internalization is likely to be necessary for tau reduction in primary neurons [72]. Unknown Ab endosomal escape mechanisms for the clearance of tau after endocytosis may exist, as the receptor-mediated endocytosis of Ab was found to be enhanced in the presence of tau aggregates in neurons. Moreover, after anti-tau Abs were internalized through certain mechanisms and were bound to cytosolic tau, the resultant complexes bound to TRIM21, known as not only E3 ubiquitin ligase but also as FcR, via the Fc domain, were subjected to ubiquitin-mediated degradation [73]. Therefore, the internalization or formation of tau-Ab complexes in the neuronal cytosol and successive ubiquitin degradation via TRIM21 as FcR can also be a promising strategy for intraneuronal tau clearance using anti-tau Abs. Modes of Fc-FcR interactions depend on Ab features, FcR types, and binding tau species.

#### 2.8.2. Transportation across the BBB

Several Abs, such as DMR7, SKT82, and other anti-tau IgG molecules, have been reported to be successfully transported across the BBB in in vivo tests [61]. However, in general, Abs are unable to cross the BBB due to their large size and hydrophilic features. Interestingly, although RmAb158, a mAb against the soluble Aβ protofibrils, was reported to be transported into the central periventricular area across the blood-CSF barrier, some were transported into the cortex through damaged BBB, as a result of leakage [62]. BBB dysfunction is known to occur in AD [74]. mAbs in the bloodstream have a long half-life due to salvation based on FcRn-mediated endocytosis and successive recycling back to the blood stream at the endothelial cells without lysosomal degradation. They may be leaked gradually and specifically into the brain through the injured tight junction of the capillary endothelial cells at the BBB due to their high molecular size, similar to how nanoparticles with payloads accumulate in cancer tissues through the loose tight junctions of endothelial cells via the EPR effect [8]. However, delivery based on Ab leakage at a disordered BBB depends on the conditions of dysfunction, and may not be effective or appropriate for prophylactic use before AD pathogenesis. Therefore, more strategic approaches need to be developed. Potential methodologies are reviewed below. In addition, aducanumab has been reported to dose-dependently lower Aβ plaques in AD and evoke BBB disruption. By contrast, anti-Aβ mAbs that did not evoke BBB disruption did not lower Aβ plaques. Thus, the transendothelial mechanism of aducanumab is thought to be leakage through BBB disruption, most likely due to vasogenic edema or cerebral microhemorrhage [2]. In general, mAbs cannot cross the BBB, and aducanumab has the unique ability to cross the BBB by inducing BBB disruption.

##### Role of FcRn of the Endothelium at the BBB

FcRn [11] plays a vital role in transferring Abs across enterocytes in the small intestine, podocytes, renal proximal tubular cells of the kidney, and syncytiotrophoblasts of the placenta. In contrast, FcRn endocytoses Abs and recycle them back, via a process known as salvation, in the vascular endothelial cells and hepatocytes of the liver. When IgG Abs are transported to the brain across the endothelium through injured tight junctions, they are likely to be delivered from the brain to the systemic circulation through reverse transcytosis based on FcRn of the endothelial cells at the BBB [75]. This is consistent with the features of general endothelial cells that recycle back IgG molecules in endosomes to the systemic circulation based on FcRn, which results in extending the IgG half-life without being degraded in lysosomes. Furthermore, bystander Abs are internalized into endosomes based on pinocytosis, subsequently bind to FcRn via acidification and are recycled back into the bloodstream. As a result, a delivery strategy using FcRn did not achieve this purpose. Thus, in contrast to mAb delivery across the small intestinal epithelium, mAbs must be transcytosed using other receptor-mediated transcytosis systems to cross the endothelial cells at the BBB.

##### Transendothelium Based on Receptor-Mediated Transcytosis

Receptor-mediated transcytosis occurs across endothelial cells at the BBB using the insulin receptor (InsR) and transferrin receptor (TfR), as well as other receptors [9]. This indicates that substances conjugated with ligands that bind to such receptors can be delivered across the BBB [76]. TfR and anti-TfR Ab are often used as ligands for transendothelium substance delivery. When ligand-TfR affinity is moderate, the ligand is liberated from TfR by endosome maturation. Subsequently, free ligands are released into the brain through fusion between the basolateral membrane and endosome. On the other hand, when ligand-TfR affinity is high, ligand-TfR complexes are degraded in lysosomes without being released into the brain as a result of the sorting process. In an in vitro assay using SV40-immortalized adult rat brain endothelial cells (SV-ARBEC), among anti-TfR, rat bivalent Ab OX26 variants, namely OX2676 and OX26108 with medium affinity to TfR, were distributed in early endosomes and exhibited enhanced transcytosis, whereas OX265 with high affinity was distributed in late endosomes and lysosomes [77]. Moreover, in in vitro live imaging using bEND.3 cells, bispecific Ab against TfR with low affinity and γ-secretase (BACE1) did not reduce the TfR level, while that with high affinity reduced it by distributing to lysosomes and being degraded there together with TfR [78]. Interestingly, bispecific Ab against TfR and BACE1 intravenously administered in vivo in monkeys was reported to cross the BBB and reduce brain in an Aβ and TfR-dependent manner [79]. A similar system can be applied for bispecific Abs against TfR and tau species.

#### 2.8.3. Plausible Design of mAbs to Clear Tau

**Figure 12:**
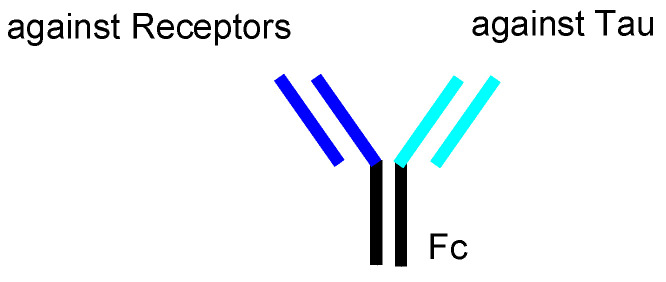
The structure of bispecific IgG against receptors and tau species.

**Figure 13:**
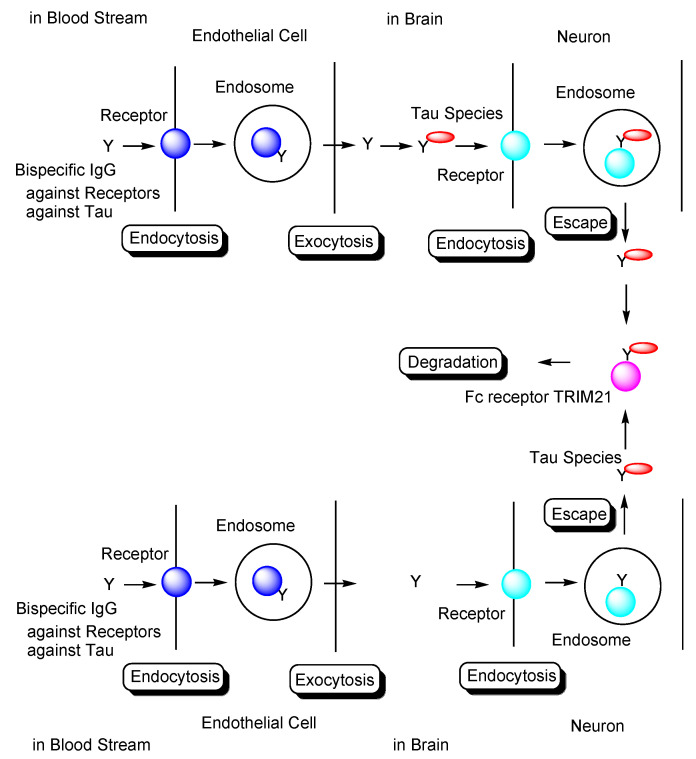
Tau degradation by the ubiquitin-proteasome pathway through Fc receptor-mediated endocytosis in neurons using bispecific Abs against tau and receptors. TRIM21 stands for tripartite motif-containing protein 21.

**Figure 14:**
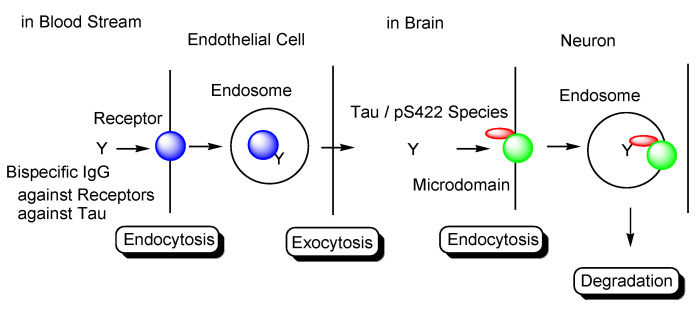
Tau/pS422 species lysosomal degradation through membrane microdomain-mediated endocytosis in neurons using bispecific Abs against tau/pS422 and receptors.

In general, mAbs cannot enter the brain across the BBB. Thus, bispecific mAbs with Fc domain targeting not only tau species in the brain but also receptors inducing transcytosis at the BBB represent a promising molecular design (Figure 12). With respect to the intended trajectory: (i) intravenously administered well-designed bispecific mAbs could cross the BBB into the brain through receptor-mediated transcytosis using suitable receptors such as TfR, bind to arbitrary extracellular tau species, and be degraded with captured tau proteins in lysosomes after Fc receptor-mediated endocytosis into microglia (Figure 2); (ii) they can cross the BBB into the brain through receptor-mediated transcytosis using suitable receptors such as TfR, be internalized into neurons through Fc receptor-mediated endocytosis, bind to arbitrary cytosolic tau species after endosomal escape through unknown mechanisms, and be degraded by TRIM21-associated ubiquitin degradation (Figure 13). In particular cases, (iii) they can cross the BBB into the brain through receptor-mediated transcytosis using suitable receptors, such as TfR, bind to tau species, such as tau/pS422, exposed on lipid rafts of neuronal plasma membrane, be internalized into neurons through lipid raft-mediated endocytosis, and be degraded in lysosomes (Figure 14).

The choice of pathological tau species for use as antigens remains elusive due to complex heterogeneity of tau. Therefore, anti-tau Abs targeting tau species, such as tau oligomers, need to be screened through repetitive in vivo experiments using animal models of AD.

#### 2.8.4. Combination Therapies

The pathology of AD is widely considered to progress in a highly complex manner. Therefore, combination therapies represent potentially successful strategies for enhancing the therapeutic efficacy of AD treatment. This is supported by the fact that, to date, single-modality therapies have failed to show effectiveness against AD.

While senile plaques have been reported to be eliminated by active immunotherapy using AN-1792, dementia resulting from AD proceeded in this case, with no neurons firing there, according to Mach’s principle of perception. In other words, nerve regeneration did not occur in response to treatment. Most likely, glial cells occupied the empty space after neuronal death. Based on these results, there is a need to pave the way for nerve regeneration in AD pathology. PD symptoms were reported to improve by injecting the fetal brain into the brains of PD patients aged 60 and younger [80]. Furthermore, umbilical cord matrix stem cell (UCMSC) transplantation was reported to improve the rotational behavior of PD model rats [81]. However, the sampling and use of fetal brain cells or UCMSCs is made difficult by their limited numbers and associated ethical issues. Mesenchymal stem cells (MSCs), which were first found in 2001, represent an alternative for differentiation into neurons. Autologous adipose-derived MSC therapy has been used for the treatment of several neurological diseases, including brain stem hemorrhage and cerebral infarction, and neurodegenerative diseases, such as amyotrophic lateral sclerosis (ALS), AD, PD, and neuropathy. In general, stem cells have the ability to spontaneously migrate to sites of injury, nidus, and regeneration [82], via the so-called homing process, and are able to cross the BBB after their intravenous administration.

Even though tau oligomers of mild-to-moderate dementia patients are eliminated by anti-tau mAbs, Aβ oligomers may not only continue to injure neurons but also enhance the production of pathological tau oligomers as seeds, and must therefore also be eliminated. AN-1792-induced anti-Aβ Abs have been reported to eliminate senile plaques [16], although they did not eliminate all Aβ oligomers [17]. Aβ can be cleared by AN-1792-induced anti-Aβ Abs outside the BBB via the dynamic equilibrium of Aβ between the brain and blood plasma [83]. Thus, Aβ-targeting vaccines may be effective for AD therapy.

**Figure 15:**
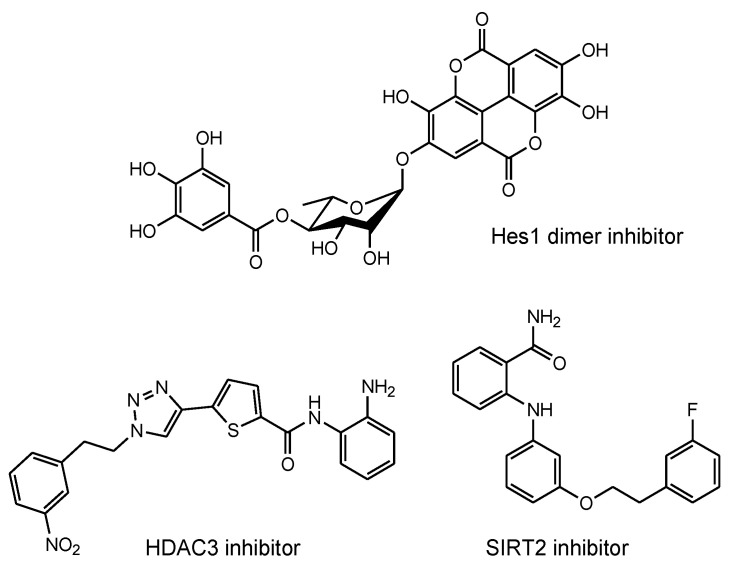
Structures of neurodegenerative disease modulators.

Combination therapy using three agents, namely anti-tau oligomer and anti-TfR bispecific mAbs, AN-1792, and MSCs, could be an extraordinarily effective and economical method for the treatment of mild-to-moderate dementia in AD patients. During and after therapy, sustained recovery can be verified using magnetic resonance imaging (MRI) against Aβ and tau. When AN-1792 elicits acute meningoencephalitis, other Aβ-targeting vaccines can be utilized. Furthermore, low-molecular compounds, such as Hes1 dimer inhibitors for neural stem cell (NSC) differentiation [84] and histone deacetylase 3 (HDAC3) inhibitors [85] and SIRT2 inhibitors [86] for neurodegenerative diseases (Figure 15), can be used in combination with immunotherapy.

## 3. Conclusions

The development of drugs for the treatment of AD has been made difficult not only due to impermeability of drugs into the brain via the BBB [9], but also due to the complex mechanisms of AD pathogenesis, wherein the pathological mechanisms of Aβ and tau are known to play crucial roles. Almost all clinical trials using Aβ modulators have failed their respective stages, with aducanumab, anti-insoluble Aβ fibrils, and anti-soluble Aβ oligomer mAb being approved by the FDA in 2021. It has been revealed that the pathologies of Aβ and tau initially progress independently, only beginning to exert their influence on each other after some time. Subsequently, the pathology of AD reaches a point of no return [4]. NFTs composed of tau rather than senile plaques composed of Aβ are correlated with AD pathogenesis [5]. It has been suggested that once certain types of tau seeds are formed, AD pathology progresses via these seed species. Accordingly, the amyloid hypothesis has been modified based on the results of clinical trial and novel findings. Tau species are thought to spread from cell to cell and be duplicated based on seeds in a prion-like manner [25]. Thus, the elimination of toxic tau seed species using specific Abs represents a promising strategy for the treatment of AD. Nonetheless, although clinical trials using anti-tau Abs have been performed, none of these have managed to reach phase 3, due to the fact that the BBB impedes the entry of anti-tau Abs into the brain. Therefore, an alternative drug design needs to be developed. Intravenously administered bispecific Abs against tau and receptors that induce receptor-mediated transcytosis in capillary endothelial cells across the BBB may represent a solution to the aforementioned trajectory issues. Furthermore, as a tau elimination mechanism, (i) tau-Ab complexes may be internalized into microglia through FcR-mediated endocytosis and degraded in lysosomes (Figure 2); (ii) anti-tau Abs may be internalized into neurons through FcR-mediated endocytosis, capture cytosolic tau species after endosomal escape, and finally be degraded by the ubiquitin pathway after binding to TRIM21 as FcR and E3 ubiquitin ligase (Figure 13); (iii) Ab complexes with tau species, such as tau/pS422, which exist on lipid rafts of neuronal plasma membrane, may be internalized into neurons through lipid raft-mediated endocytosis and degraded in lysosomes (Figure 14). Salvation by FcRn in endothelial cells can lengthen the half-life of intravenously administered bispecific Abs.

The complexity of AD pathology is so high that single-modality therapies have been widely found to be ineffective. Therefore, combination therapies have been suggested to increase the therapeutic efficacy of AD treatments. The combination of bispecific Abs against tau and receptors at capillary endothelial cells, Aβ modulators, such as AN-1792 as vaccine, and stem cells, such as MSCs, via intravenous administration are potential candidates. Furthermore, low-molecular-weight compounds may be used in combination with these treatments. Aducanumab is known to cross the BBB by disturbing it. Thus, combination therapy using aducanumab, anti-tau Abs, and MSCs represents an alternative treatment. In the field of neuroscience, qualia are defined as sensory consciousness by which a person recalls “what a certain thing is like” in the brain. When dementia as a result of AD is treated via nerve cell regeneration, whether qualia are restored remains unclear, since they are formed through individual experiences. Nonetheless, medicinal chemists and pharmaceutical scientists hope to improve the quality of life of AD patients by creating innovative immunotherapy approaches using well-designed antibodies targeting AD-relevant tau species.

## Data Availability

ClinicalTrials.gov (accessed on 25 December 2021) Identifier can be found at https://clinicaltrials.gov/ (accessed on 25 December 2021).
